# Newborn Screening in Saudi Arabia: Brief History, Current Practice, and Future Direction

**DOI:** 10.3390/ijns12020035

**Published:** 2026-05-13

**Authors:** Ahmed H. Mujamammi

**Affiliations:** Clinical Biochemistry Unit, Department of Pathology, College of Medicine, King Saud University, Riyadh 11461, Saudi Arabia; amujamammi@ksu.edu.sa

**Keywords:** national newborn screening program, dried blood spot, tandem mass spectrometry, quality assurance, ethical challenges

## Abstract

The Saudi Arabia National Newborn Screening (NBS) program is a pillar of public health, offering timely detection of treatable, life-threatening, or disabling conditions in neonates. This comprehensive review critically examines the current laboratory diagnostic practices employed for metabolite analysis within this program. It focuses primarily on biochemical NBS conducted via dried blood spot testing and evaluates the methodologies, technical challenges, and stringent quality assurance measures that underpin successful screening. This review examines the critical role of tandem mass spectrometry, sample integrity protocols, and the establishment of robust cutoff values. Furthermore, this review explores persistent challenges such as false-positive and false-negative results, ethical and logistical hurdles in global implementation, and the transformative potential of recent advancements, including the integration of genomics and high-resolution metabolomics. In addition, this review explores the future of the program, highlighting the transformative potential of high-resolution metabolomics and the integration of genomic sequencing to ensure early diagnosis and intervention.

## 1. Introduction

Newborn screening (NBS) in the Kingdom of Saudi Arabia serves as a critical public health initiative designed to detect certain congenital disorders shortly after birth. While the foundational principles of NBS trace back to the Wilson and Jungner criteria, the Saudi Arabian program has evolved into a sophisticated national system aimed at mitigating the high prevalence of inborn errors of metabolism (IEM) within the region [1,2]. This program is a government-funded initiative, ensuring that all newborns delivered in the hospitals mandated by the public health authority (Weqaya), are eligible to receive screening at no cost to their families [3,4]. Currently, the program utilizes dried blood spot (DBS) collection to screen for a comprehensive panel of disorders [1,4,5]. Unlike general reviews of the field, this article provides a detailed examination of the operational and laboratory framework specific to Saudi Arabia. We address key logistical elements, including the centralized or regional laboratory structure, the courier-based specimen transport systems used to maintain sample integrity, and the specific annual screening volume and coverage percentages achieved across the Kingdom’s diverse regions [1,2,6]. Furthermore, this paper clarifies the vital distinction between initial population-based screening and the subsequent diagnostic confirmation process. While screening is a proactive measure to identify at-risk newborn, it is distinct from “confirmatory testing”—such as urine organic acid (UOA) analysis or specialized amino acid profiles—which is used to validate presumptive positives and provide a definitive diagnosis at designated medical centers. By focusing on the unique laboratory practices and quality management protocols specific to the Saudi Arabian context, this work aims to provide a definitive record of the program’s current state and its technical trajectory.

## 2. Methods

This review was conducted through a structured search of scientific literature addressing newborn screening programs in Saudi Arabia and related laboratory methodologies. Relevant articles were identified through electronic databases including PubMed, Scopus, google scholar and Web of Science.

Search terms included newborn screening, Saudi Arabia, dried blood spot (DBS), tandem mass spectrometry (MS/MS), IEM, and metabolomics. Publications describing the implementation, clinical outcomes, and laboratory methodologies of the Saudi national newborn screening program were prioritized.

Additional information was obtained from reports of the Saudi Ministry of Health (MOH) and publications from major national screening centers. Studies focusing on diagnostic methods, screening performance, and epidemiology of screened disorders were reviewed to provide a comprehensive overview of the current screening framework.

## 3. Results

### 3.1. History and Evolution of NBS: Global Foundation and Saudi Arabian Program Development

NBS programs were first implemented in the 1960s and have since become a fundamental component of public health [7]. These programs were initially designed to test congenital disorders shortly after birth that, if left undetected or untreated, could lead to disability or death [8]. The unequivocal goal of NBS is early detection of these conditions before they become symptomatic, thereby allowing for swift therapeutic intervention [9]. The foundational method for this process is biochemical NBS performed by DBS testing, which remains the global gold standard. NBS emerged in the 1960s with the introduction of the dried blood spot test developed by Guthrie, guided by the screening principles of Wilson and Jungner [10].

The Saudi Arabian NBS program has transitioned from a limited pilot to a sophisticated, government-funded national health mandate. The initial program was established prior to 1980 in collaboration with the International Atomic Energy Agency (IAEA) by providing expertise and offering guidance on screening protocols to initially detect congenital hypothyroidism (CH) [11]. While the NBS program of the Saudi MOH began with the first pilot cord blood screening in the 1980s, the scientific groundwork for expanded screening was laid in the 1990s at King Faisal Specialist Hospital and Research Centre (KFSH&RC) [12]. The pioneering research by Rashed and colleagues at KFSH&RC established the technological foundation for Saudi Arabia’s newborn screening by proving that automated electrospray MS/MS could simultaneously diagnose a vast array of metabolic disorders from a single blood spot. This breakthrough provided a strategic, high-throughput solution to the Kingdom’s unique challenge of high regional disease prevalence, effectively ending the “one-test, one-disorder” era. Ultimately, this landmark work served as the primary catalyst for evolving local pilot projects into a comprehensive national mandate that has since screened millions of newborns and set a benchmark for public health across the Middle East [13]. This was further advanced by the introduction of 96-well microplate batch processing and computerized flagging algorithms, which transitioned the technology into a high-throughput system capable of the speed and scale required for national screening [14]. By 1999, their research solidified the clinical application of these methods, demonstrating a life-saving capacity to detect over 30 conditions and providing the final scientific justification for the Saudi MOH to adopt expanded NBS as a national standard [14,15].

In 2007, Saadallah and Rashed contextualized the Saudi Arabian experience by highlighting how high regional consanguinity rates necessitated an expanded screening panel to address a significantly higher incidence of autosomal recessive metabolic disorders. It documented the successful transition of the program from a specialized research project into a comprehensive national health mandate, serving as a regional blueprint for the Middle East and North Africa. Ultimately, this work solidified Saudi Arabia’s role as a leader in neonatal public health by demonstrating the clinical and economic necessity of high-throughput MS/MS screening in the region [16]. These local technological advancements directly facilitated the major national transition between 2004 and 2005 with the formal introduction of MS/MS, which substantially enhanced testing capabilities and paved the way for the launch of the national NBS program in 2005. From 2005 to 2012, there was a focused effort to scale up laboratory capacity across the Kingdom [1]. Following this expansion, the period from 2012 to 2018 was focused on quality assurance and test expansion. The most recent phase, spanning from 2018 to 2025, has focused on advanced laboratory integration, emphasizing standardization, rapid data communication, and clinical referral pathways for time-critical conditions [5]. Looking toward the future, the program is poised to integrate genomic sequencing and personalized metabolic medicine, ensuring that Saudi Arabia remains at the forefront of global newborn screening innovation [17].

### 3.2. Critical Role of Expanded NBS in Saudi Arabia

The NBS program in Saudi Arabia has grown significantly with the aim of identifying a comprehensive panel of conditions early in life. In Saudi Arabia, the implementation of an expanded NBS program is a critical public health necessity dictated by a unique genetic landscape where the incidence of IEM is reported to be 4-to-5-fold higher than that of Western populations [2]. This elevated prevalence is largely attributed to high rates of consanguinity, with regional studies indicating consanguineous marriage rates between 50% and 80%, which significantly increases the risk of autosomal recessive disorders [5,18]. Consequently, the Saudi MOH recently updated the national NBS panel in 2024 to include 20 specific disorders, ranging from organic acidurias like propionic aciduria to endocrine disorders [5]. This specialized scope is essential for the early detection and management of these conditions, effectively preventing irreversible neurological damage and reducing the substantial socioeconomic burden on the national healthcare system [5]. A comprehensive list of the 23 screened disorders and their clinical categories is provided in Table 1.

The methods used for screening NBS panels rely heavily on high-throughput multiplex technologies applied to DBS samples. Most IEMs, specifically amino acidopathies, organic acid disorders, and fatty acid oxidation defects, are screened using MS/MS. MS/MS is a powerful platform that rapidly and simultaneously measures primary and secondary metabolites (such as amino acids and acylcarnitines) related to multiple disorders in a single assay, making it highly efficient [19,20]. For non-metabolic disorders, other assay platforms are employed. For instance, CH is screened using immunoassays to measure thyroid-stimulating hormone (TSH), and congenital adrenal hyperplasia (CAH) is screened by measuring 17-hydroxyprogesterone (17-OHP), often utilizing an automated benchtop analyzer designed for DBS using samples, such as a Genetic Screening Processor (GSP^®^, Revvity, Turku, Finland) or equivalent instrumentation or similar time-resolved fluorescence immunoassay platforms for the initial detection of these markers [1,19] (Table 1). The Saudi National NBS program’s strategic design is underpinned by the empirical metrics and validation parameters detailed in Table 2. These localized data points justify the Kingdom’s expanded screening scope, which is necessitated by a unique genetic landscape. As shown in the table, the incidence rates of metabolic disorders in Saudi Arabia significantly exceed global averages, a nuance primarily attributed to regional consanguinity requiring specifically calibrated diagnostic thresholds to maintain high accuracy within the population.

### 3.3. Infrastructure and Operational Logistics of the Saudi National NBS Program

#### 3.3.1. Advancing Newborn Screening: The Saudi Arabian Integrated Model

The Saudi Arabian NBS program operates under a centralized laboratory model designed to ensure high-quality, standardized testing across the Kingdom’s vast geographical area. The primary reference hub is the Newborn Screening Laboratory at KFSH&RC in Riyadh, which serves as the national center for advanced biochemical and molecular analysis [2]. To manage the increasing birth rate and geographical diversity, the MOH has integrated regional screening clusters such as the Riyadh second health cluster which act as satellite units for primary collection and initial processing [5]. These laboratories participate in rigorous external quality assurance (EQA) programs, including those provided by the College of American Pathologists (CAP) and the U.S. Centers for Disease Control and Prevention (CDC) newborn screening quality assurance program (NSQAP), ensuring that local metabolic cutoffs and analytical performance meet international benchmarks [1,19].

Logistically, the program utilizes a dedicated medical courier system rather than standard postal services to transport DBS specimens from over 270 maternity hospitals to the regional (cluster) laboratories. This system is strictly regulated to maintain climate control and ensures that samples reach the analytical hub within 24 to 48 h of collection, a critical factor for time-sensitive disorders like GALT and MSUD [1]. As a result of these logistical improvements and the government’s mandate under Vision 2030, the national coverage rate is being expanded to reach over 95% of all live births in the Kingdom [5]. The program is fully funded by the government, providing universal, cost-free screening to all citizens and residents, thereby removing financial barriers to early diagnosis and intervention.

**Figure 1:**
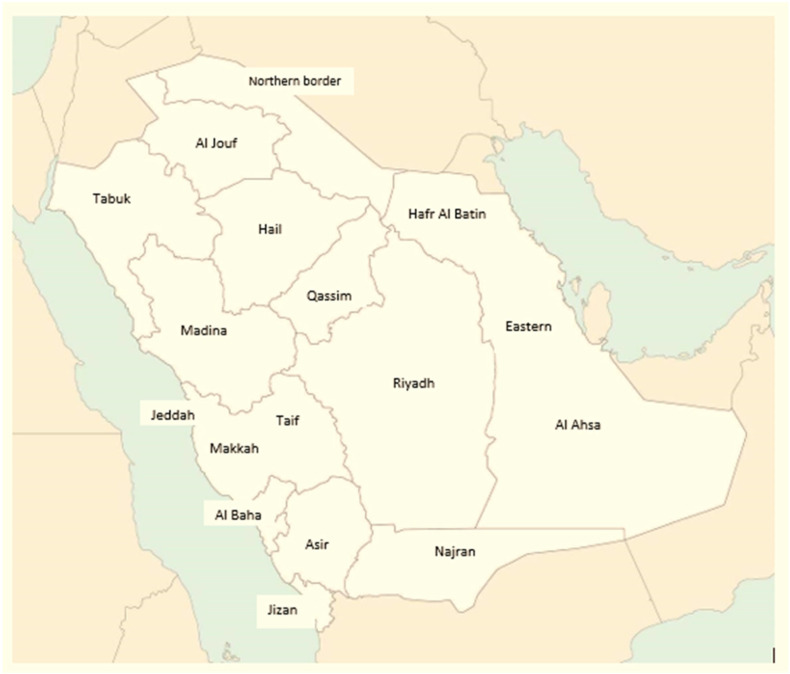
National distribution of the health clusters supporting the Saudi national newborn screening program followed by the MOH.

The screening process begins with the collection of DBS samples from newborns at birthing hospitals 24–72 h after birth, which are then transported to Weqaya newborn screening laboratories. The laboratories within the Kingdom fall within three categories, including laboratories under the MOH, other governmental laboratories such as those under military hospitals and university hospitals, and the third are private laboratories. All of these laboratories are involved independently in the processing of NBS samples and generating results. All results from second and third categories, by regulation, are required to provide the results along with the samples to the Central lab under MOH, that is Weqaya for archiving. NBS in Saudi Arabia is centrally managed by the Saudi NBS Program under the Public Health Authority (Weqaya), which serves as the national reference laboratory and coordinating center. Analysis is primarily performed using tandem mass spectrometry (LC-MS/MS) and immunoassay-based platforms. Within this centralized framework, the Weqaya reference laboratory is responsible for standardizing testing protocols, establishing and validating analyte cutoffs, ensuring quality assurance through proficiency testing, and training laboratory staff and healthcare providers. The program currently achieves over 95% coverage of the approximately 500,000–600,000 annual births in the Kingdom. While all MOH hospitals refer testing to Weqaya, screening also occurs in some private and other governmental facilities, with all delivery departments mandated to comply with national regulations regarding mandatory NBS and follow-up. The system is supported by health clusters in Saudi Arabia, there are 20 clusters that are distributed among the country as follow: three in Riyadh, Qassim, Eastern, AlAhsa, Hafr AlBatin, Makkah, Madinah, two in Jeddah, AlTaif, Hail, Tabuk, Al Jouf, Northern Boarders, Aseer, Najran, Jazan and Al Baha (Figure 1).

The regional screening laboratory acts as the primary trigger for the follow-up system; once a critical value is detected, the lab immediately notifies a regional coordinator to ensure the infant is seen within 24–48 h. Reflex testing performed on the initial DBS specimen is strictly part of the screening algorithm, whereas confirmatory diagnosis requires independent diagnostic testing via plasma, urine, enzymatic assays, or molecular genetic analysis. These confirmatory tests and subsequent management are handled by metabolic centers, which typically manage a recall rate of 0.5–1%, ultimately identifying confirmed disorders in approximately 1 in 1000–2500 births. A multidisciplinary team of metabolic pediatricians, clinical biochemical geneticists, genetic counselors, dietitians, and specialized laboratory scientists provides care. However, challenges remain regarding the limited number of metabolic specialists in certain regions, prompting the increased use of telemedicine between tertiary centers and regional hospitals. Program efficacy and national data are maintained centrally through the Weqaya NBS Program database, which collects laboratory results, confirmatory outcomes, and long-term follow-up data. Key performance indicators monitored include number of newborns screened, coverage rate, specimen quality indicators, turnaround time, recall rate, positive predictive value, confirmed case numbers, treatment initiation time [1,5,6].

Analyte cutoffs are determined in collaboration with program experts and are periodically reviewed based on population-specific data, validation studies, and international benchmarking. Adjustments occur when screening sensitivity requires optimization, new disorders are added, or new technologies are implemented. Furthermore, the Saudi NBS coordinates the training and monitoring of birthing facilities, ensuring maternity staff are proficient in DBS collection and conducting periodic audits to maintain specimen quality.

#### 3.3.2. Laboratory Diagnostic Framework: Implementation and Validation in the Saudi Context

The operational success of the Saudi National NBS program relies on a transition from generalized screening protocols to a refined, population-specific diagnostic framework. This evolution is defined by localized validation studies, the integration of multitier analytical modalities, and a strategic focus on resolving the biochemical ambiguities inherent in a highly consanguineous population.

The confirmatory framework in Saudi Arabia follows a tiered diagnostic protocol that bridges centralized laboratory science with regional clinical expertise. Once a presumptive positive is identified via primary MS/MS or GSP^®^, reflexive testing is initiated. For aminoacidopathies, confirmation is performed via quantitative plasma amino acid (PAA) analysis using ion-exchange chromatography; for organic acidemias, urine organic acid (UOA) profiles are obtained via gas chromatography-mass spectrometry (GC-MS) [22]. Following biochemical confirmation, the program incorporates reflexive molecular testing to identify population-specific founder mutations, which is particularly vital for resolving diagnostic ambiguity in consanguineous families [22,23]. While the specialized testing is centralized, the clinical follow-up and long-term management are decentralized through a network of regional metabolic centers at tertiary hospitals (e.g., King Fahad Medical City), where multidisciplinary teams provide life-saving metabolic management and genetic counseling [1,5].

#### 3.3.3. Optimized DBS Logistics and Local Validation of GSP^®^

The reliability of the Saudi program begins with the integrity of the DBS sample, which serves as the primary sample for all initial screens [24,25]. The spots are completely air-dried at ambient temperature and shielded from direct heat or sunlight, which is a critical step in stabilizing temperature-sensitive metabolites. All the standard collection and stabilization protocols are followed as per international standards to ensure metabolite detection. The Saudi initiative for detection of metabolites from DBS sample is distinguished by its rigorous, data-driven foundation that uses the GSP^®^ system. This system specifically identifies individual metabolites such as GALT, biotinidase deficiency (BTD), 17-OHP, G6PD and TSH as part of NBS [24,25]. This extensive research utilized the GSP^®^ to establish Saudi-specific cutoff values and analyte ratios for CAH, and BTD as an example. The cutoff was calculated by taking population-based percentiles from local cases, the program optimized the GSP^®^’s analytical performance, achieving a positive predictive value (PPV) exceeding 80% and a false-positive rate of less than 0.04% for the majority of analytes [19].

In Saudi practice, the GSP^®^ is critical for detecting thyroid dyshormonogenesis—a group of autosomal recessive defects in thyroid hormone synthesis that occur at a disproportionately higher rate in the Kingdom due to consanguinity [26,27]. The Saudi program utilizing these specific GSP^®^ metrics in DBS is being used as an initial screening that can be validated by measuring serum TSH levels or molecular sequencing in newborns that have failed the primary TSH screen [5,28].

#### 3.3.4. MS/MS Application and the Saudi Metabolic Landscape

MS/MS serves as the definitive workhorse of the Saudi laboratories, providing the high-throughput multiplexing necessary to screen for dozens of disorders in a single run [22,29]. The program strategically utilizes MS/MS to screen for three principal classes of inherited metabolic disorders: amino acidemias (e.g., PKU and MSUD), fatty acid oxidation disorders (e.g., MCADD and VLCADD), and organic acid species identified via acylcarnitine conversion [30,31]. To maintain accuracy across the diverse geography of the Kingdom, the integration of stable isotope internal standards is used to compensate for technical variability, including fluctuations in extraction efficiency and collected blood volume [32].

#### 3.3.5. Resolving Biochemical Ambiguity: Reflexive Molecular Workflows

A hallmark of the Saudi Arabian NBS program is its pioneered integration of second-tier molecular workflows as part of an enhanced screening protocol [23,33]. In the Saudi context, molecular confirmation is often mandatory to resolve biochemical ambiguities, noted in tandem MS, GSP^®^ and electrophoretic pattern for hemoglobinopathies, encountered in a population with a high genetic load and specific founder mutations [19,33,34]. This ambiguity arises not from phenotypic variability but from the elevated background prevalence of heterozygous carriers which inflate the rate of borderline biochemical signals and therefore require resolution. Local implementation studies have demonstrated that employing targeted genetic analysis immediately following an abnormal biochemical screening allows for the rapid identification of specific Saudi founder mutations, such as those seen in the PCCA and PCCB genes for Propionic Acidemia [23]. Research at KFSH&RC has shown that this integrated clinical pathway achieves a molecular diagnostic yield of over 90% for newborns referred from the national program [35]. This synergy between biochemical and molecular testing represents a refined laboratory protocol calibrated to the Kingdom’s Vision 2030 goals, significantly reducing parental distress by minimizing unnecessary recall [5,36].

#### 3.3.6. Non-TMS Modalities in NBS: UOA and Enzyme Assays

For cases requiring secondary validation, the program utilizes UOA analysis and specialized enzyme assays. While initial MS/MS screening identifies at-risk neonates, the definitive diagnosis of organic acidurias relies on the identification of pathognomonic metabolites through UOA profiling [37,38,39]. In Saudi clinical practice, this allows for the differentiation of disorders with overlapping markers, such as confirming Glutaric Aciduria Type 1 through elevated 3-hydroxyglutaric acid [20,40]. Additionally, lysosomal storage disorders (LSDs) have been identified to have a high prevalence in the Kingdom. Based on their increasing prevalence, Saudi specialized centers have designed pilot studies that utilize enzyme assays to detect LSDs, like Pompe or Fabry disease [1]. By measuring residual enzyme activity against population-specific thresholds established in Saudi tertiary centers, clinicians can provide a critical diagnostic indicator for these recessive conditions [41,42].

#### 3.3.7. Quality Management and Interpretation Challenges

**Figure 2:**
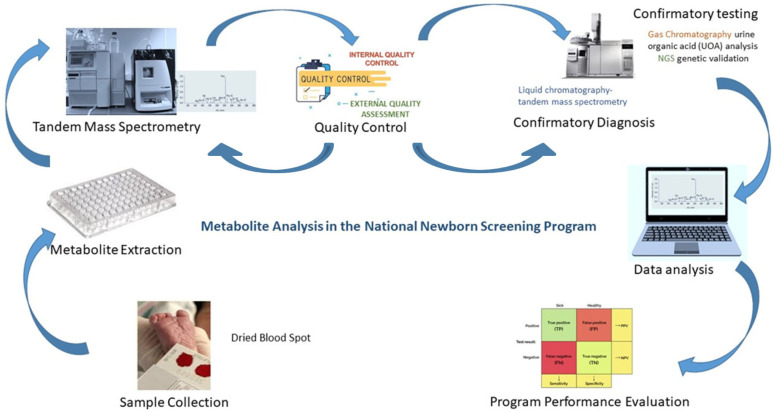
Representation of the procedural flow for metabolite analysis in newborn screening.

The ethical responsibility of the NBS program demands a robust quality management system. Saudi laboratories utilize specialized quality control materials prepared on filter paper to replicate the unique patient matrix [43,44]. Participation in external quality assessment (EQA) schemes ensures inter-laboratory comparability across the national network [45]. The interpretation of these results remains the most complex stage of the process. Saudi clinicians must account for confounding biological variables such as gestational age, birth weight, and total parenteral nutrition that influence metabolite levels [46,47]. To improve specificity, the program has moved toward the use of analyte ratios (e.g., the phenylalanine/tyrosine ratio for PKU) and sophisticated interpretive algorithms that integrate patient demographics to assign risk scores [19,48]. This comprehensive approach ensures that the Saudi NBS program functions not merely as a technical implementation, but as a calibrated clinical system optimized for the Kingdom’s unique demographic needs. Figure 2 illustrates the sequential steps involved in metabolite analysis within the NBS program.

### 3.4. Logistical and Ethical Challenges in NBS

The success of NBS hinges on robust logistical systems and continuous ethical oversight. Logistically, the process demands rapid execution, with the entire “time-to-result” and “time-to-treatment” ideally occurring within the first 1–2 weeks of life [48]. Failures at chokepoints, such as delayed sample collection or slow transport and processing, can lead to irreversible damage and pose a constant challenge, particularly in resource-limited or geographically diverse areas such as in cases of CH, MSUD, GALT and BTD similar to other countries [49]. A key challenge is the trade-off between sensitivity and specificity that is seen commonly in enzymatic assays carried out for patients with organic acidemias and enzymatic disorders [1,50]. Although high sensitivity can ensure that the affected newborns are not missed, a high false-positive screening rate can cause significant parental anxiety (the “tyranny of the positive result”) and incurs substantial healthcare costs for unnecessary follow-ups [51]. Conversely, although rare, false-negative screens are catastrophic system failures that can lead to death or irreversible onset of disease symptoms, demanding a continuous protocol review [52]. Ethically, the expanding NBS panel continuously debates the Wilson and Jungner principle, particularly the inclusion of disorders in which the long-term benefit of early intervention is not definitively proven, or those with variable expressivity [53]. The introduction of genomic screening adds further complexity, raising concerns about the incidental discovery of adult-onset conditions that require clear ethical frameworks and informed consent processes [54].

### 3.5. Emerging Technologies and Future Directions

The field of NBS is undergoing a rapid technological transformation, moving toward increased throughput, enhanced accuracy, and personalized diagnostics. The limitations of traditional targeted MS/MS are overcome by untargeted high-resolution mass spectrometry (HRMS), which utilizes instruments such as Orbitraps (Thermo Fisher Scientific, Waltham, MA, USA) to measure the mass-to-charge ratio of thousands of metabolites with extreme accuracy [55,56,57]. This practice is followed in the MOH laboratories and is being incorporated in other hospitals [4]. This untargeted approach will allow for the discovery of new biomarkers and offers a deeper view of the neonatal metabolome, enabling the simultaneous screening of numerous disorders and better differentiation of disease variants [58]. However, the vast data output from HRMS necessitates sophisticated bioinformatics tools and machine learning algorithms for complex data processing and statistical modeling to translate metabolic signatures into clinical diagnoses [36]. The integration of next generation sequencing represents a significant future direction for newborn screening, as gene-based methods have the potential to directly address biochemical ambiguity by reducing false-positive rates and substantially boosting the positive predictive value (PPV). Moreover, the inherent capability of NGS to simultaneously screen for hundreds of single-gene disorders makes it an increasingly feasible and cost-effective technology for comprehensive NBS [59].

A major advancement in the Saudi program is the integration of genomics as a reflexive second-tier screening tool [60]. Under this protocol, a positive initial biochemical screen can be rapidly followed by targeted gene sequencing using the original DBS sample. While its utility in CH is specifically targeted toward the high prevalence of dyshormonogenesis in the Kingdom, it remains exceptionally effective for IEMs and conditions with nonspecific or variable biochemical markers where genetic etiology is certain.

Ultimately, this synergy of biochemical and molecular testing significantly improves the PPV, drastically reducing the false-positive rate and the associated parental distress [61]. The ultimate future direction of NBS is personalized care, which may involve developing algorithms for individualized cutoffs that adjust metabolite action limits based on the specific physiological variables of the newborns (e.g., gestational age or feeding status) [36]. Further improvements will result from seamless integration with electronic health records, facilitating longitudinal tracking, and improving time-critical follow-up efficiency [62]. Finally, the potential development of point-of-care testing using rapid miniature analytical systems may decentralize testing and accelerate the diagnostic timeline for critical disorders in remote or low-resource settings despite challenges in achieving laboratory-grade accuracy [63].

## 4. The Future Trajectory

Looking ahead, the Saudi NBS program is moving toward a more personalized and technologically integrated model of care. The next decade of innovation is expected to focus on the routine integration of high-resolution metabolomics and expanded genomic sequencing to further refine diagnostic accuracy and resolve biochemical ambiguities in the Saudi population. These advancements are closely aligned with the broader healthcare transformation goals of Vision 2030, which emphasizes precision medicine and standardized clinical referral pathways [64]. By leveraging these emerging technologies, the program aims to not only detect disorders earlier but also to offer tailored therapeutic interventions, ensuring that Saudi Arabia remains at the global forefront of NBS and personalized metabolic health [33,65].

## 5. Conclusions

The Saudi NBS program has evolved from localized pilot projects into a sophisticated, government-funded public health mandate that ensures universal, cost-free screening for over 95% of all live births. This expansion is a critical response to a unique genetic landscape where the incidence of IEM is 4-to-5-fold higher than in Western populations, primarily driven by high regional consanguinity rates. By utilizing a centralized laboratory model anchored by MS/MS and the GSP^®^, the program effectively manages a comprehensive panel of over 20 disorders. The integration of reflexive second-tier molecular workflows and specialized confirmatory testing ensures diagnostic accuracy and reduces parental distress by minimizing false-positive screening. As the program aligns with the precision medicine goals of Saudi Vision 2030, the future trajectory involves incorporating high-resolution metabolomics and expanded genomic sequencing to further refine personalized neonatal care and maintain the Kingdom’s position at the forefront of global screening innovation.

## Figures and Tables

**Table 1:** Comprehensive national newborn screening panel in Saudi Arabia: disorders and screening analytes.

Disease Category	Disorder	Screening Analytes		
		Primary *	Secondary *	Other Metabolic Markers
Disorders of amino acid metabolism	Phenylketonuria	Phe	Phe/Tyr ratio	Increased urine phenyl ketoacids
	Maple Syrup Urine Disease (MSUD)	Leu ± Ileu	Val	PAA: Allo-Ileu
	Citrullinemia Type I (Cit-I)	Cit	-	PAA: Arg UOA: OA
	Argininosuccinic Aciduria (ASA)	Cit	-	PAA: ASA UOA: OA
	Homocystinuria	Meth	-	PAA: Hcy
	Tyrosinemia Type I (Tyr-I)	SA ± Tyr	-	Phe; Meth; increased urine Tyr metabolites
Organic Acidemias	Propionic Acidemia	C3	C3/C2	UOA: PA; 3-OH PA; MCA; TG
	Methylmalonic Acidemia (MMA)	C3	C3/C2	UOA: MMA; 3-OH PA; MCA; TG
	Isovaleric Acidemia (IVA)	C5	-	UOA: 3-OH IVA, IVG
	Glutaric Acidemia Type I (GA-I)	C5-DC	-	UOA: GA; 3-OH GA
	3-Methylcrotonyl-CoA Carboxylase Deficiency (3MCC)	C5-OH	-	UOA: 3-OH IVA; 3-MCG
	3-Hydroxy-3-Methylglutaryl-CoA Lyase Deficiency	C5-OH	C6-DC	UOA: 3-OH IVA; 3-HMG; 3-MGA; 3-MG
	Beta-Ketothiolase Deficiency	C5-OH	C5:1	UOA: 2M3BHA; MAA; TG
Disorders of Fatty Acid Oxidation	Carnitine Uptake Deficiency	C0	-	Low total plasma carnitine
	Medium-chain acyl-CoA dehydrogenase deficiency	C8	C6; C10; C10:1; C8/C10; C8/C2	Increased medium-chain dicarboxylic acids
	Very long-chain acyl-CoA dehydrogenase deficiency	C14:1	C14; C14:2; C16; C18:1; C14:1/C2	Increased medium/long-chain dicarboxylic acids
Endocrine & Other Disorders	CH	TSH	-	Serum TSH
	CAH	17-OHP	-	Serum 17-OHP
	Galactosemia (GALT)	GALT	Galactose	Enzyme activity
	Biotinidase Deficiency (BTD)	BTD	-	-
Red Cell Enzyme Disorders	Glucose-6-phosphate Dehydrogenase Deficiency(G6PD)	G6PD	-	Whole blood, Enzyme activity
Hemoglobinopathies	Sickle Cell Disease	HGBE **	-	Whole blood, HEBE **
	Thalassemia	HGBE **	-	Whole blood, HEBE **

* DBS, dried blood spot (DBS) (Primary Screening); PAA, Plasma Amino Acids (confirmatory); UOA, Urine Organic Acids (confirmatory). ** varies. 17-OHP = 17-hydroxyprogesterone, 2M3BHA = 2-methyl-3-hydroxbutyric acid, 3-HMG = 3-hydroxy-3-methylglutaric acid, 3-MCG = 3-methylcrotonoylglycine, 3-MG = 3-methylglutaric acid, 3-MGA = 3-methylglutaconic acid, 3-OH GA = 3-hydroxyglutaric acid, 3-OH IVA = 3-hydrixyisovaleric acid, 3-OH PA = 3-hydroxypropionic acid, Allo-Ileu = Allo-isoleucine, Arg = Arginine, BTD = Biotinidase, C0 = Free carnitine, C10 = Decanoylcarnitine, C10:1 = Decenoylcarnitine, C14 = Tetradecanoylcarnitine, C14:1 = Tetradecenoylcarnitine, C14:2 = Tetradecadienoylcarnitine, C16 = Hexadecanoylcarnitine, C18:1 = Octadecanoylcarnitine, C2 = Acetylcarnitine, C3 = Popionylcarnitine, C5 = Isovalerylcarnitine, C5-DC = Glutarylcarnitine, C5-OH = Hydroxyisovalerylcarnitine, C5:1 = Tiglylcarnitine, C6 = Hexanoylcarnitine, C6-DC = Methylglutarylcarnitine, C8 = Octanoylcarnitine, Cit = Citrulline, GA = Glutaric acid, GALT = Galactose-1-phosphate uridyltransferase, HGEB = Hemoglobin Electrophoresis, Hcy = Homocysteine, Ileu = Isoleucine, IVG = Isovalerylglycine, Leu = Leucine, MAA = 2-methylacetoacidic acid, MCA = Methylcitric acid, Meth = Methionine, MMA = Methylmalonic acid, OA = Orotic acid, PA = Propionic acid, PAA = Plasma Amino Acid, Phe = Phenylalanine, SA = Succinylacetone, TG = Tiglylglycine, TSH = Thyroid-Stimulating Hormone, Tyr = Tyrosine, UOA = Urine Organic Acid, Val = Valine.

**Table 2:** Evidence-based summary on the key metrics and validation parameters of Saudi Arabia National Newborn Screening (NBS) Program.

Metric	Findings	Clinical Significance and Validation	References
Panel scope	23 disorders	Includes aminoacidopathies, organic acidemias, FAO and endocrine.	[1,21]
Consanguinity Rate	56.7–67%	Primary driver of high autosomal recessive disease prevalence.	[5,18]
Overall Incidence (IEM)	1:891 to 1:1043	4-to-5-fold higher than Western populations; justifies expanded scope.	[1,5,19]
Sample Size (Validation)	*n* ≥ 74,000 newborns	8-year longitudinal study (2013–2020) used for Saudi-specific cutoffs.	[2]
Analytical Precision	PPV > 80% **	Achieved through population-based percentiles (99.5–99.9th)	[19]
False-Positive Rate	<0.04 *	Optimized for GSP^®^ and MS/MS analyte	[19]
Most Common IEMs	Propionic Acidemia	Local incidence (~1:10,000) significantly exceeds global rates.	[1,5]

FAO, Fatty Acid Oxidation; IEM, inborn errors of metabolism; PPV, positive predictive value; GSP^®^, Genetic Screening Processor; MS/MS, Tandem Mass Spectrometry; * false-positive rate of <0.04% applies for majority of analytes and does not represent the cumulative false-positive rate across the entire screening panel. ** Positive Predictive value (PPV) applies for majority of disorders except for endocrine disorders, red cell enzyme disorders and hemoglobinopathies.

## Data Availability

The original contributions presented in this study are included in the article. Further inquiries can be directed to the corresponding author.

## Abbreviations

The following abbreviations are used in this manuscript:

NBS	Newborn Screening
DBS	Dried Blood Spot
MS/MS	Tandem Mass Spectrometry
IEMs	Inborn Errors of Metabolism
NGS	Next- Generation Sequencing
CH	Congenital Hypothyroidism
CAH	Congenital Adrenal Hyperplasia
GSP^®^	Genetic Screening Processor
TRFIA	Time-Resolved Fluorescence Immunoassay
TSH	Thyroid-Stimulating Hormone
17-OHP	17-Hydroxyprogesterone
QMS	Quality Management System
IQC	Internal Quality Control
EQA	External Quality Assessment
